# Intersecting social determinants of health among patients with childcare needs: a cross-sectional analysis of social vulnerability

**DOI:** 10.1186/s12889-024-18168-8

**Published:** 2024-02-29

**Authors:** Anisha P. Ganguly, Kristin S. Alvarez, Sheryl R. Mathew, Virali Soni, Suman Vadlamani, Bijal A. Balasubramanian, Kavita P. Bhavan

**Affiliations:** 1 Center of Innovation and Value, Parkland Health, Dallas, TX USA; 2 Department of Internal Medicine, University of Texas Southwestern Medical Center, Dallas, TX USA; 3 School of Medicine, University of Texas Southwestern Medical Center, Dallas, TX USA; 4 Department of Epidemiology, Human Genetics, and Environmental Sciences, The University of Texas Health Science Center at Houston, School of Public Health, Houston, TX USA; 5 Institute for Implementation Science, The University of Texas Health Science Center at Houston, School of Public Health, Houston, TX USA; 6 Health Equity Fellow, Parkland Health, 5200 Harry Hines Blvd, Dallas, TX 75235 USA

**Keywords:** Social determinants of health, Childcare, Social vulnerability index, Intervention, Geospatial analysis

## Abstract

**Introduction:**

Access to childcare is an understudied social determinant of health (SDOH). Our health system established a childcare facility for patients to address childcare barriers to healthcare. Recognizing that social risk factors often co-exist, we sought to understand intersecting social risk factors among patients with childcare needs who utilized and did not utilize the childcare facility and identify residual unmet social needs alongside childcare needs.

**Methods:**

We conducted a cross-sectional analysis of patients who enrolled in the childcare facility from November 2020 to October 2022 to compare parameters of the Social Vulnerability Index (SVI) associated with the census tract extracted from electronic medical record (EMR) data among utilizers and non-utilizers of the facility. Overall SVI and segmentation into four themes of vulnerability (socioeconomic status, household characteristics, racial/ethnic minority status, and housing type/transportation) were compared across utilizers and utilizers. Number of 90th percentile indicators were also compared to assess extreme levels of vulnerability. A sample of utilizers additionally received a patient-reported social needs screening questionnaire administered at the childcare facility.

**Results:**

Among 400 enrollees in the childcare facility, 70% utilized childcare services and 30% did not. Utilizers and non-utilizers were demographically similar, though utilizers were more likely to speak Spanish (34%) compared to non-utilizers (22%). Mean SVI was similar among utilizers and non-utilizers, but the mean number of 90th percentile indicators were higher for non-utilizers compared to utilizers (4.3 ± 2.7 vs 3.7 ± 2.7, *p* = 0.03), primarily driven by differences in the housing type/transportation theme (*p* = 0.01). Non-utilizers had a lower rate of healthcare utilization compared to utilizers (*p* = 0.02). Among utilizers who received patient-reported screening, 84% had one unmet social need identified, of whom 62% agreed for additional assistance. Among social work referrals, 44% were linked to social workers in their medical clinics, while 56% were supported by social work integrated in the childcare facility.

**Conclusions:**

This analysis of SDOH approximated by SVI showed actionable differences, potentially transportation barriers, among patients with childcare needs who utilized a health system-integrated childcare facility and patients who did not utilize services. Furthermore, residual unmet social needs among patients who utilized the facility demonstrate the multifactorial nature of social risk factors experienced by patients with childcare needs and opportunities to address intersecting social needs within an integrated intervention. Intersecting social needs require holistic examination and multifaceted interventions.

**Supplementary Information:**

The online version contains supplementary material available at 10.1186/s12889-024-18168-8.

## Introduction/background

Social determinants of health (SDOH) are foundational drivers of health outcomes [1]. There is strong and rapidly growing evidence demonstrating the inextricable linkage of social, economic, and environmental factors with health and healthcare outcomes. While there is an established relationship between health and socioeconomic drivers like housing [2], food security [3], and transportation [4], access to safe, reliable childcare remains a less recognized and less studied SDOH [5]. However, for families with children and particularly female caregivers, access to childcare impacts our economy and society, a reality amplified during the COVID-19 pandemic as women disproportionately left the workforce due to disruptions in childcare [6, 7]. Similarly, access to childcare has significant implications on public health. Early data, including major findings from the 2017 Kaiser Family Foundation (KFF) Women’s Health Survey, have shown that childcare needs played a significant role in parents’ access to healthcare [8].

Lack of childcare has substantially impacted healthcare access in our health system, Parkland Health (hereafter, “Parkland”). Located in Dallas County, TX, the second least-insured large city in the country [9], Parkland is a safety-net health system that serves a largely low-income, underinsured, racially diverse patient population [10]. Women seeking medical services in our system have reported lack of childcare to be a leading factor for missed appointments; a cross-sectional survey of 336 reproductive-age women seen at Parkland outpatient clinics found that lack of childcare (53%) surpassed other barriers to accessing healthcare, including lack of transportation (33%) and lack of insurance (25%) [11]. Survey participants additionally reported delaying an average of 3.7 appointments per year due to these barriers [11].

In response to these findings, Parkland partnered with a local non-profit organization to develop Annie’s Place, a health system-integrated childcare center for patients. All patients are eligible to utilize the childcare center free of charge while attending a scheduled appointment for anything from preventive care to medical emergencies. Annie’s Place is staffed by licensed childcare professionals along with play therapists, thereby collaboratively providing cognitive and behavioral support for children along with meeting parents’ childcare needs. Since opening mid-pandemic in November 2020, Annie’s Place has provided childcare for more than 600 patients accessing healthcare services.

Among the early literature exploring childcare access as a SDOH, childcare needs are often presented alongside other logistical barriers to care, such as financial strain, transportation, and insurance [12–14]. Recognizing that social risk factors rarely occur in isolation, holistic, intersectional perspectives are needed to address health inequities [15]. Similarly, comprehensive understanding of intersecting unmet social needs with childcare needs is necessary to inform childcare interventions and implementation of such interventions. Prior social sciences literature has demonstrated linkages between childcare barriers and health-related social needs including financial instability [16], homelessness [17], food insecurity [18, 19], and lack of transportation [19].

We sought to better understand the role of social risk factors occurring alongside childcare needs and identify residual unmet social needs that may inhibit engagement in Parkland’s health system-integrated childcare facility. In this study, we leveraged geospatial data to approximate differences in social risk factors by comparing domains of social vulnerability among patients who utilized the childcare facility and those who did not utilize the facility despite enrolling in childcare services. We hypothesized that women who did not utilize the childcare facility would have increased indicators of social vulnerability, indicating unmet social needs that could limit engagement in the childcare intervention. Furthermore, we sought to screen women who utilized the childcare facility for residual unmet social needs with an in-person screening questionnaire to characterize co-existing social needs and streamline linkage to resources through contact in the childcare facility.

## Methods

### Health system-integrated childcare facility

Parkland integrated no-cost childcare for patient’s during appointments by partnering with a community-based organization (CBO) that specializes in caring for children of patients with unmet childcare needs [20]. Patients for whom childcare is a barrier to appointments or other healthcare services (e.g., laboratory, imaging, pharmacy encounters) may utilize the drop-off childcare facility. Patients with childcare are connected to no-cost childcare by self-referral, social work resource connection, or by clinical personnel, including electronic referral, patient portal messaging, brochures, clinic signage, and after-visit summary printouts. Once linked to no-cost childcare, patients are enrolled for services within 24–48 h. After designation as a childcare enrollee, patients may utilize childcare services for appointments or other health needs.

### Study population and time period

Patients with children enrolled in no-cost childcare between November 2020 and October 2022 were included. Indication of a childcare need was defined by enrollment in the childcare facility. Enrollment in the facility may be initiated by patient self-referral, provider-initiated referral, and electronic medical record (EMR) patient portal questionnaire. Patients may self-refer and enroll directly with the facility using contact information publicized in clinical after visit summaries (AVS), signage posted in the health system, brochures disseminated in patient waiting areas, the health system website, and the CBO website. If a patient indicates a childcare need in a clinical encounter, a provider can place a referral to the facility, which prompts the facility to contact the patient and complete enrollment. Patients who indicate a childcare need via the EMR patient portal questionnaire are subsequently contacted by the childcare to complete enrollment.

The exposure of interest among the population of enrollees was utilization. The utilizer cohort was defined as patients who completed at least one childcare appointment within 6 months of enrollment. The non-utilizer cohort did not use childcare services within 6 months of enrollment.

### Geospatial analysis of social vulnerability

Enrolled patients were geocoded based on their address location within the 2020 census tract map. The patients within each census tract were distributed based on density (size of marker) within a specific census tract. Geospatial determinants of health (GDOH), which is used to define the geospatial drivers of health with an emphasis on factors that vary by place [21], was used to measure parameters of social vulnerability and thereby approximate social risk factors relevant to patients enrolled in childcare services. The 2020 CDC/ATSDR Social Vulnerability Index (SVI) [22] was used to categorize enrolled patients into different levels of overall vulnerability based on four themes: socioeconomic status, household characteristics, racial/ethnic minority status, and housing type/transportation, comprised of 16 indicators of social vulnerability (Supplemental Table 1). Similar methodology has been leveraged to approximate social risk factors in a variety of clinical contexts, including obesity [23], surgical outcomes [24], and COVID-19 infection [25]. Notably, SVI was utilized by the Geospatial Research, Analysis, and Services Program (GRASP) for equitable allocation of COVID-19 vaccines to marginalized communities [26]. The CDC/ATSDR SVI metrics used for analysis rank census tracts for the entire United States against each other based on the four themes to generate a relative vulnerability score. Tract rankings are percentile based, with values ranging from 0 to 1 (100th percentile), where higher values signify greater vulnerability relative to the normalized population, which in this analysis included United States (US) percentiles and Texas state percentiles. In addition, themes and individual indicators flagged as being in the top 90th percentile in the US and Texas were also utilized for comparison of patients associated with areas with highest levels of social vulnerability.

### Patient-reported social needs

To measure social needs besides childcare among patients linked to the childcare facility intervention, a sample of enrolled utilizers received a patient-reported social needs questionnaire administered by the onsite social worker. The survey was administered from June 2021 to October 2022. The sample was administered to all patients utilizing the childcare facility during this time period. The EMR embedded questionnaire, adapted from the Centers for Medicare & Medicaid Services (CMS) Accountable Health Communities Health-Related Social Needs Screening Tool, consisted of seven questions pertaining to socioeconomic, housing and transportation needs, and an additional question regarding linkage to social services [27]. These questionnaire responses were incorporated into the health system’s EMR and social services specific to the need identified were offered. Each question was mapped to two of the four CDC/STSDR SVI identified themes of socioeconomic status or housing type and transportation. Question responses were considered positive for social needs if answered as ‘Somewhat hard,’ ‘Hard,’ or ‘Very hard,’ ‘Sometime true,’ or ‘Often true,’ or ‘Yes’ to any of the applicable survey questions. If two surveys were administered during the study timeframe, then the first one was used in the analysis. The remaining variables of racial/ethnic minority status and household characteristics were obtained through the EMR.

### Study outcomes and statistical analysis

Baseline characteristics were compared among enrolled Annie’s Place utilizers and non-utilizers. The primary outcome assessed was the overall and four thematic CDC/ATSDR SVIs compared between enrolled utilizers and non-utilizers. To further discern differences in rightward skewed percentiles, number of indicators flagged in the 90th percentile were also compared across the two cohorts. Secondary outcomes included healthcare utilization of utilizers and non-utilizers, defined by appointment scheduling rate and attendance rate. Additionally, the patient-reported and EMR captured unmet social needs among the sample of surveyed utilizers was represented with descriptive statistics. Categorical data for primary and secondary outcomes utilized the χ2 test. Continuous variables were all non-parametric and evaluated using the Kruskal Wallis test. *P*-values < 0.05 were considered statistically significant. All statistical analysis was performed with Python 3.7 (Python Software Foundation). This study was approved by the University of Texas Southwestern Institutional Review Board.

## Results

**Fig. 1:**
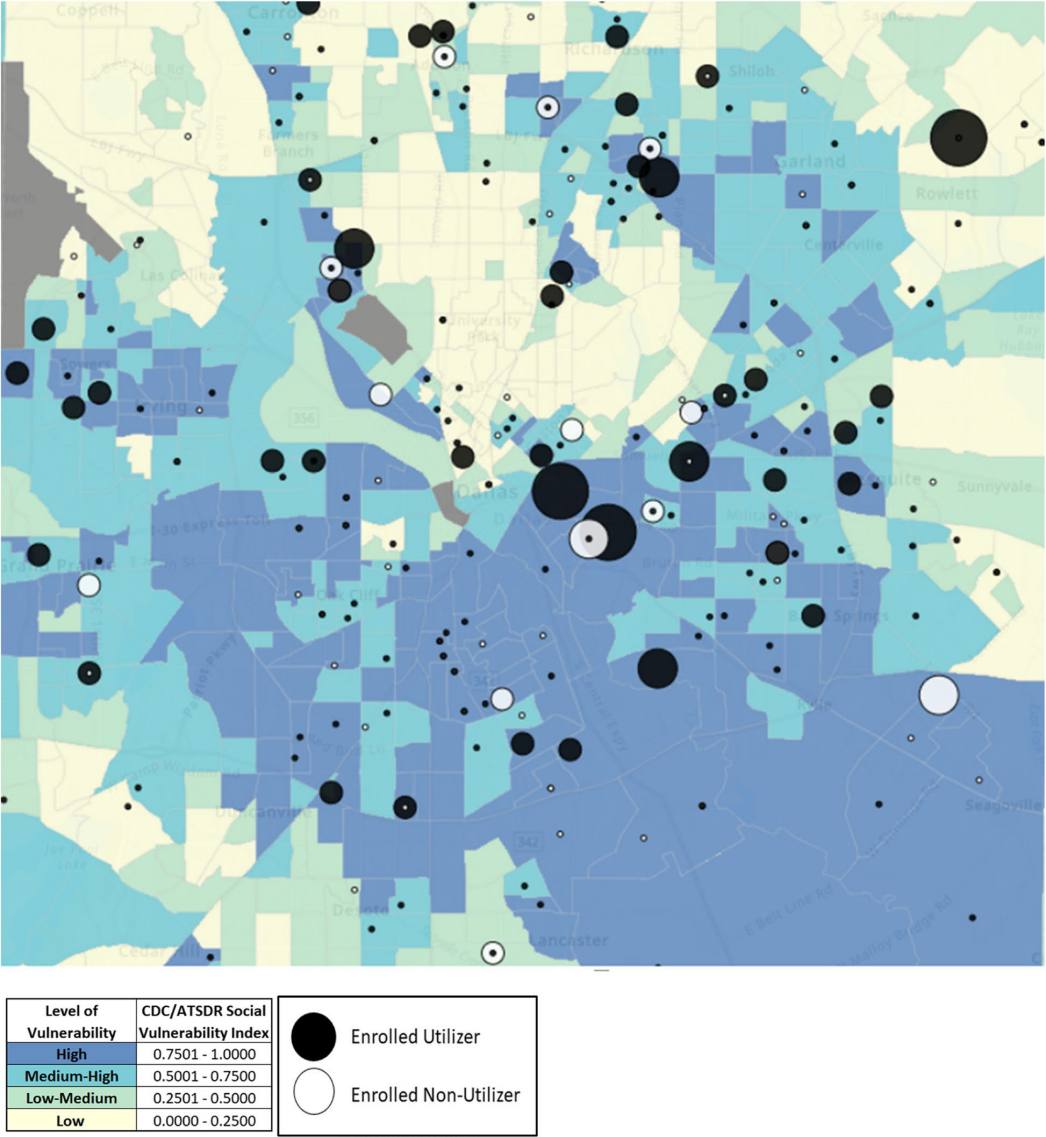
Distribution of childcare facility enrolled utilizers and non-utilizers addresses and census tract-level social vulnerability index normalized to US-level percentiles

Four hundred patients enrolled in no-cost childcare services from 11/2020 to 10/2022. Patients enrolled in services were primarily female (96%), identified as Hispanic (53%) and Non-Hispanic-Black (35%) with a mean age of 32.4 (Table 1). Figure 1 shows the distribution of enrolled utilizers’ and non-utilizers' communities of residence with the GDOH identified. Of these, 279 (70%) completed at least one childcare appointment within six months of enrollment. There was a greater percentage of Spanish-speakers among the utilizer cohort (34%) than the non-utilizer cohort (22%). No differences in race/ethnicity, year enrolled, or the number or age of enrolled children were found between cohorts.

**Table 1:** Baseline characteristics of childcare facility utilizers and non-utilizers

	**All Enrolled (*N* = 400)**	**Utilizer (*N* = 279)**	**Non-Utilizer (*N* = 121)**	***P*-value**
**Mean Age (SD)**	32.4 (8.8)	32.9 (9.1)	31.1 (8.0)	0.03
**Female Gender (%)**	384 (96.0%)	269 (96.4%)	115 (95.0%)	0.71
**Ethnicity/Race (%)**				0.06
Hispanic	221 (52.5%)	158 (56.6%)	52 (43.0%)	
Non-Hispanic Black	139 (34.7%)	87 (31.2%)	52 (43.0%)	
Non-Hispanic White	34 (8.5%)	25 (9.0%)	9 (7.4%)	
Asian	8 (2.0%)	4 (1.4%)	4 (3.3%)	
Other	9 (2.3%)	5 (1.8%)	4 (3.3%)	
**Preferred Language**				0.04
English	268 (67.0%)	176 (63.1%)	92 (76.0%)	
Spanish	122 (30.5%)	96 (34.4%)	26 (21.5%)	
Other	10 (2.5%)	7 (2.5%)	3 (2.5%)	
**Year Enrolled**				0.74
2020	25 (6.3%)	16 (5.7%)	9 (7.4%)	
2021	177 (44.3%)	126 (45.2%)	51 (42.1%)	
2022	198 (49.5%)	137 (49.1%)	61 (50.4%)	
**Mean Number of Children Enrolled (SD)**	1.54 (0.76)	1.59 (0.81)	1.41 (0.62)	0.09
**Mean Age of Children Enrolled (SD)**	2.87 (1.92)	2.95 (2.00)	2.69 (1.71)	0.31
**Total Clinic Appts**	13.6 (15.6)	14.8 (17.4)	10.4 (9.3)	0.02

There was a similar overall SVI among utilizers and non-utilizers (0.72 ± 0.25 and 0.75 ± 0.24, *P* = 0.28) and similarity across all four SVI themes (Table 2). Non-utilizers trended towards high level of overall SVI, though not reaching the threshold of statistical significance (*p* = 0.16). The number of 90th percentile vulnerability indicators were higher in non-utilizers across all four themes, with 3.7 ± 2.7 indicators/patient among utilizers and 4.3 ± 2.7 indicators/patient among non-utilizers (*p* = 0.03). When segmented into comparison across individual themes, only the difference in the theme of housing type/transportation was statistically significant (0.73 ± 0.82 indicators/patient among utilizers and 0.95 ± 0.81 indicators/patient among non-utilizers, *p* = 0.01), though there was a trend towards differences in socioeconomic status. The same comparison of SVI percentiles normalized within the state of Texas was conducted (Supplemental Table 2). The difference in the housing type/transportation theme persisted when comparing 90th percentiles normalized at the state level. Further segmentation of the housing type/transportation theme into 4 discrete indicators demonstrated a trend towards differences in transportation and crowded housing (Supplemental Table 3), though not reaching the threshold of statistical significance (*p* = 0.17 and 0.13, respectively).

**Table 2:** Comparison of SVI normalized to US percentiles among childcare facility utilizers and non-utilizers

	**All Enrolled (*N* = 400)**	**Utilizer (*N* = 279)**	**Non-Utilizer (*N* = 121)**	***P*-value**
**Overall SVI (SD)**	**0.73 (0.25)**	0.72 (0.25)	0.75(0.24)	0.28
**Socioeconomic Status Theme Index (SD)**	**0.76 (0.24)**	0.75 (0.24)	0.78(0.23)	0.23
**Household Characteristics Theme Index (SD)**	**0.65 (0.28)**	0.65 (0.28)	0.65(0.29)	0.76
**Racial Ethnic Minority Theme Index (SD)**	**0.82 (0.14)**	0.81 (0.15)	0.83(0.13)	0.36
**Housing type/Transportation Theme Index (SD)**	**0.57 (0.27)**	0.56 (0.27)	0.59(0.25)	0.44
**Overall Level**^**a**^				
High	**252 (63.0%)**	169 (60.6%)	83 (68.6%)	0.16
Medium–High	**81 (20.3%)**	63 (22.6%)	18 (14.9%)	0.10
Low-Medium	**41 (10.3%)**	27 (9.7%)	14 (11.6%)	0.69
Low	**26 (6.5%)**	20 (7.2%)	6 (5.0%)	0.55
**Any Indicator in 90th Pct (%)**	**356 (89%)**	243 (87.1%)	113 (93.4%)	0.09
**Number of 90th Pct Indicators/patient (SD)**	**3.92 (2.7)**	3.72 (2.7)	4.3 (2.7)	0.03
**Number of 90th Pct Indicators/patient (SD)**				
Socioeconomic Status	**1.72 (1.39)**	1.66 (1.39)	1.88 (1.39)	0.11
Household Characteristics	**1.12 (0.97)**	1.08 (0.98)	1.20 (0.95)	0.21
Racial Ethnic Minority	**0.28 (0.45)**	0.27 (0.44)	0.31 (0.46)	0.45
Housing type/Transportation	**0.80 (0.82)**	0.73 (0.82)	0.95 (0.81)	0.01

^a^Low defined as SVI 0.0 to 0.25, low-medium defined as SVI 0.2501 to 0.5, medium–high defined as SVI 0.5001 to 0.75, high defined as SVI 0.7501 to 1

Utilizers had a statistically higher number of clinic visits scheduled compared with non-utilizers (14.8 ± 17.4 vs. 10.4 ± 9.3, *p* = 0.02) as well as a statistically higher completion rate for clinic appointments (73% vs. 64%, *p* = 0.02). Nineteen patients (four in the utilizer group and 15 in the non-utilizer group) did not have a clinic appointment scheduled within 6 months of childcare enrollment.

One hundred and thirty-four childcare utilizers received in-person social need questionnaires (Table 3). The response rate was 100%. Most utilizers identified at least one social need (84%), and the highest social vulnerability theme identified via survey was socioeconomic status (79% screened positive for financial strain), followed by housing type/transportation themed vulnerability (35.8% positive respondents). Demographics of surveyed utilizers captured from the EMR identified racial/ethnic minority status themed social vulnerability in 92% of utilizers, followed by 42% positive for household characteristics themed social vulnerability.

**Table 3:** In-person social needs questionnaire administered to utilizers of no-cost childcare

Survey Question	Answer Choices^a^	SDOH Need (*N* = 134)
**How hard is it for you to pay for the very basics like food, housing, medical care, and heating?**^b^	*Not hard at all*	9 (7%)
	*Not very hard*	34 (25%)
	*Somewhat hard*	55 (41%)
	*Hard*	14 (10%)
	*Very hard*	17 (13%)
	*Patient refused*	4 (3%)
**Within the past 12 months**		
…you worried that your food would run out before you got money to buy more.^b^	*Never true*	66 (49%)
	*Sometimes true*	51 (38%)
	*Often true*	16 (12%)
	*Patient refused*	1 (1%)
…the food you bought just didn’t last and you didn’t have money to get more.^b^	*Never true*	71 (53%)
	*Sometimes true*	44 (33%)
	*Often true*	16 (12%)
	*Patient refused*	3 (2%)
…was there a time when you were not able to pay the mortgage or rent on time?^b^	*Yes*	57 (42%)
	*No*	73 (54%)
	*Patient refused*	2 (1%)
…was there a time when you did not have a steady place to sleep or slept in a shelter (including now?)^c^	*Yes*	13 (10%)
	*No*	119 (88%)
	*Patient refused*	0 (0%)
…has lack of transportation kept you from medical appointments or from getting medications?^c^	*Yes*	39 (29%)
	*No*	95 (70%)
	*Patient refused*	0 (0%)
…has lack of transportation kept you from meetings, work, or getting things needed for daily living?^c^	*Yes*	37 (28%)
	*No*	96 (71%)
	*Patient refused*	1 (1%)
**Does your child have health insurance?**	*Yes*	117 (87%)
	*No*	14 (10%)
	*Patient refused*	3 (2%)

^a^Question responses were considered indicative of SDOH needs if answered as ‘Somewhat hard’, ‘Hard’, or ‘Very hard’, ‘Sometime true’, or ‘Often true’, or ‘Yes’ to any of the applicable survey questions

^b^Mapped Questions for Socioeconomic Status CDC/ATSDR SVI Theme

^c^Mapped Questions for Housing type & transportation CDC/ATSDR SVI Theme

Among the 113 patients who screened positive for social needs with the in-person screening questionnaire, 70 (62%) agreed to additional assistance for identified needs. Of these 70 patients, 100% were referred for social work consultation. Among social work referrals, 31 patients (44%) were linked to social workers in primary care and specialty care clinics where they were receiving medical care, and 39 patients (55.7%) received social work support through the childcare center itself.

## Discussion

In this study, we examined the intersection of multiple co-existing social risk factors among patients unmet childcare needs in the context of engagement with a childcare intervention. Our analysis compared census tract-level geospatial indicators of social vulnerability associated with patients who utilized a health system-integrated childcare center and patients who were referred and enrolled in childcare services but ultimately did not utilize them. Overall, both cohorts of patients were associated with high levels of social vulnerability. Further analysis revealed that on average, non-utilizers were more likely to be associated with extreme levels of social vulnerability, defined by 90th percentile indicators, compared to utilizers. Segmentation of SVI themes demonstrated that this difference was primarily driven by differences in parameters of transportation access and congregate housing.

These findings have important implications for understanding residual, unmet social needs that impact access to healthcare services, as well as interventions integrated in the health system, such as our system-integrated childcare facility. The study findings illustrate that while the childcare facility is successful in engaging populations with demonstrated high levels of social vulnerability (current utilizers), there remains an outstanding population with quantifiably higher levels of social vulnerability who reported childcare needs and interest in the childcare center by virtue of enrollment yet may remain unable to engage with the intervention. These findings may represent a spectrum of social vulnerability, in which utilizers and non-utilizers both exhibit high levels of vulnerability, yet non-utilizers may exist beyond the spectrum threshold of engagement with the childcare intervention due to competing social risk factors.

Characterization of the residual non-utilizer population is essential for refining implementation of the childcare intervention to ultimately increase uptake. Given that this population has decreased engagement in the health system (as evidenced by lower rates of appointment scheduling and attendance) and thereby decreased health information including screening for social needs, geospatial factors were examined to approximate the social vulnerability of the non-utilizer population. Such geospatial approaches have been utilized to predict primary care needs and inform primary care access for marginalized populations [28, 29]. While prior applications of the SVI have primarily focused on characterization of disparities through the association of social vulnerability with poor health outcomes [24, 30], more recent studies have applied the SVI to inform implementation of disparity interventions [31]. Similarly, we applied the lens of social vulnerability to explore implementation gaps of an social needs intervention, recognizing the importance of competing unmet social needs in effectively addressing any single social need [32].

In this study, comparison of SVI indicators suggest disparate vulnerability associated with the underexplored population of non-utilizers. The ostensible residual social risk factors, namely transportation barriers, revealed in this analysis potentially represent actionable needs that can be navigated in the implementation of our childcare intervention. Differences in transportation access across the two cohorts provides important context to the differences in healthcare utilization, which suggests that transportation gaps similarly influence healthcare access [33] as well as access to the childcare facility. This may indicate some interaction between childcare needs and transportation barriers limiting not only appointment attendance, but decisions to even schedule appointments. Future investigation is needed to further examine the relationship between childcare needs and transportation barriers. Future improvements to our childcare facility intervention implementation may include screening for transportation barriers and if applicable, navigation to transportation resources (e.g., Medicaid transportation [34], rideshare-based medical transportation [35]) to support utilization of childcare services.

At a broader level, these findings exemplify how the confluence of multiple social risk factors interact with social support and healthcare access. As outlined in the WHO Conceptual Framework for Action on Social Determinants of Health, health inequities are the result of multiple environmental conditions, psychosocial factors, and social and economic policies, within which there is significant interplay [36]. There are increasing calls to examine SDOH with a multifactorial approach to better understand causal pathways and accordingly improve intervention design [37]. Prior research has examined the cumulative effect of multiple social risk factors on healthcare engagement and participation in health behaviors (e.g., food insecurity and financial instability [38–40], addiction and criminal justice involvement [41, 42], lack of insurance and homelessness [43, 44]). In our population of patients with reported childcare needs, we found co-existing social vulnerabilities that reflect the intersecting complexity of SDOH and the structural determinants that underly them [45].

In our sample of self-reported data from utilizers of the childcare intervention, we found that even patients who were able to engage in the childcare center continued to have high rates of social needs, including financial strain and food insecurity. This finding reflects the importance of screening for residual unmet social needs as part of any given intervention. Every patient who requested assistance with screened social needs were linked to social work for in-depth consultation, and among these patients, more than half received social work support integrated in the childcare center itself. This linkage illustrates how interventions like this health system-integrated childcare center can be leveraged to address multiple social needs in a streamlined setting.

This study has important limitations. These findings were generated in the context of a unique intervention, a health system-integrated childcare facility, which to our knowledge remains relatively uncommon in healthcare systems [20, 46]. To understand the non-utilizer population, we relied on geospatial parameters of social vulnerability in the absence of clinical or patient-reported data; the SVI serves as an approximation of social risk factors associated with place but does not provide concrete patient-level capture of social needs. The SVI does not directly measure unmet social needs, but rather measures vulnerability that can reflect differential social risk factors tied to place. Prior research has shown that community-level assessments of social risk factors may underestimate social needs when validated against patient-reported sources [47]. Geospatial data may be subject to an ecological fallacy [48], in which assumptions of individuals may be incorrectly made based on wider assessments, including neighborhood-level data sources as in this analysis [49]. Additional research is needed to collect patient-level data to understand the relationship of childcare needs related to competing social risk factors. Furthermore, the SVI is derived from measurable CDC indicators of social vulnerability and does not provide a thorough assessment of each theme examined in this study, another limitation of geospatial data sources [49]. The salient theme of housing type/transportation examined in this analysis does not provide granular information about housing and transportation as discrete social risk factors, and the variables that comprise this theme are not comprehensive. For example, the primary parameter for transportation is quantified by distribution of individuals lacking a vehicle [22], however does not assess distribution of public transportation or rideshare availability. Therefore, conclusions drawn from individual parameters necessitate further exploration to inform future SDOH screening and interventions. Strengths of this study include the diversity of the patient sample, essential for exploration of SDOH [50–52], the holistic lens of co-existing social risk factors [37], and the dual examination of geospatial and patient-reported data for a comprehensive assessment of SDOH [53, 54].

## Conclusions

We examined social vulnerability among patients enrolled in a childcare intervention to understand residual unmet social needs that may influence engagement in the intervention and healthcare in general. This investigation revealed a convergence of multiple social risk factors alongside childcare needs that underscore the cross-cutting nature of social vulnerability and the need for multifaceted approaches to social needs interventions. Future research is needed to further elucidate causal mechanisms of interacting SDOH with childcare access. Segmentation of social vulnerability revealed actionable differences among patients utilizing and not utilizing the intervention, which may inform future intervention implementation. Lastly, the childcare intervention itself served as a streamlined opportunity for additional social needs screening and linkage to support. This finding illustrates the opportunity to address multiple unmet social needs through a single streamlined intervention.

### Supplementary Information

**Supplementary Material 1. **

## Data Availability

The datasets generated and/or analysed during the current study are not publicly available due to patient privacy policies of our health system but are available from the corresponding author on reasonable request.
