# Supplementary material for: Intersecting social determinants of health among patients with childcare needs: a cross-sectional analysis of social vulnerability
Source: BMC Public Health. 2024 Feb 29;24:639. doi: 10.1186/s12889-024-18168-8 (PMC10902938; doi:10.1186/s12889-024-18168-8)
Supplement: Supplementary file 1 — Supplementary Material 1. [file 12889_2024_18168_MOESM1_ESM.docx]

**Supplemental Tables**

| Supplemental Table 1: CDC/STSDR SVI Themes and Comprised Indicators | |
| --- | --- |
|  | **CDC/ATSDR SVI** |
| **Measurement** | Ranking is based on percentiles with values between 0 and 1 (higher indicates greater vulnerability). Theme-specific and overall percentiles are used.  Low is defined as SVI 0.0 to 0.25, low-medium is defined as SVI 0.2501 to 0.5, medium-high is defined as SVI 0.5001 to 0.75, high is defined as SVI 0.7501 to 1. |
| **Socioeconomic Status** | - Below 150% poverty - Unemployed - Housing cost burden - No high school diploma - No health insurance |
| **Household Characteristics** | - Aged 65 or older - Aged 17 or younger - Civilian with a disability - Single-parent households - English language proficiency |
| **Racial/ethnic minority status** | - Hispanic or Latino (of any race) - Black and African American, Not Hispanic or Latino - American Indian and Alaska Native, Not Hispanic or Latino - Asian, Not Hispanic or Latino - Native Hawaiian and Other Pacific Islander, Not Hispanic or Latino - Two or More Races, Not Hispanic or Latino - Other Races, Not Hispanic or Latino |
| **Housing type & transportation** | - Multi-unit structures - Mobile homes - Crowding - No vehicle - Group quarters |

Supplemental Table 2. Comparison of SVI normalized to Texas percentiles among childcare facility utilizers and non-utilizers.

| **Primary outcome comparing SVI between utilizers and non-utilizers (Texas)** | | | | |
| --- | --- | --- | --- | --- |
|  | **All Enrolled (N=400)** | **Utilizer**  **(N=279)** | **Non-Utilizer (N=121)** | **p-value** |
| **Overall SVI** **(SD)** | 0.65 (0.26) | 0.64 (0.27) | 0.68 (0.25) | 0.27 |
| **Socioeconomic Status Theme Index (SD)** | 0.67 (0.26) | 0.66 (±0.27) | 0.70(±0.25) | 0.22 |
| **Household Characteristics Theme Index (SD)** | 0.57 (0.28) | 0.57 (0.28) | 0.58 (0.28) | 0.72 |
| **Racial Ethnic Minority Theme Index (SD)** | 0.68 (0.21) | 0.67 (0.21) | 0.69 (0.21) | 0.37 |
| **Housing type/Transportation Theme Index (SD)** | 0.59 (0.26) | 0.58 (0.27) | 0.61 (0.25) | 0.37 |
| **Overall Level***    High    Medium-High    Low-Medium    Low | 170 (42.5%)  127 (31.7%)  58 (14.5%)  45 (11.2%) | 111 (39.8%)  95 (34.0%)  38 (13.6%)  35 (12.5%) | 59 (48.8%)  32 (26.4%)  20 (16.5%)  10 (8.3%) | 0.12  0.17  0.55  0.28 |
| **Any Indicator in 90^th^ Pct (%)**  **Number of 90^th^ Pct Indicators/patient (SD)** | 305 (76.2%)  2.55 (2.3) | 208 (74.5%)  2.45 (2.4) | 97 (80.2%)  2.78 (2.2) | 0.28  0.08 |
| **Number of 90^th^ Pct Indicators/patient (SD)**  Socioeconomic Status  Household Characteristics  Racial Ethnic Minority  Housing type/ Transportation | 0.95 (1.15)  0.69 (0.84)  0.14 (0.35)  0.76 (0.86) | 0.92 (1.17)  0.69 (0.84)  0.12 (0.33)  0.72 (0.87) | 1.03 (1.11)  0.70 (0.85)  0.18 (0.39)  0.88 (0.84) | 0.18  0.98  0.14  0.04 |

Supplemental Table 3. Comparison of individual SVI indicators at 90^th^ percentile among utilizers and non-utilizers.

|  | **All Enrolled (N=400)** | **Utilizer**  **(N=279)** | **Non-Utilizer (N=121)** | **p-value** |
| --- | --- | --- | --- | --- |
| **Socioeconomic Status** | | | | |
| Below 150% poverty (%) | 104 (26%) | 68 (24%) | 36 (30%) | 0.32 |
| Civilian Unemployed (%) | 28 (7%) | 17 (6%) | 11 (9%) | 0.39 |
| Housing cost-burdened occupied housing units (%) | 90 (23%) | 61 (22%) | 29 (24%) | 0.74 |
| No high school diploma (%) | 192 (48%) | 127 (46%) | 65 (54%) | 0.16 |
| Uninsured (%) | 276 (69%) | 190 (68%) | 86 (71%) | 0.64 |
| **Household Characteristics** | | | | |
| 65 or older (%) | 4 (1%) | 2 (1%) | 2 (2%) | 0.75 |
| 17 and younger (%) | 146 (37%) | 97 (35%) | 49 (40%) | 0.33 |
| Disability (%) | 19 (5%) | 12 (4%) | 7 (6%) | 0.70 |
| Single-parent households (%) | 97 (24%) | 67 (24%) | 30 (25%) | 0.97 |
| Limited English (%) | 181 (45%) | 124 (44%) | 57 (47%) | 0.70 |
| **Racial and Ethnic Minority** | | | | |
| Minority Status (%) | 112 (28%) | 75 (27%) | 37 (31%) | 0.525 |
| **Housing Type/Transportation** | | | | |
| Multi-unit housing (%) | 111 (28%) | 75 (27%) | 36 (30%) | 0.64 |
| Mobile homes (%) | 22 (6%) | 14 (5%) | 8 (7%) | 0.69 |
| Crowded households (%) | 132 (33%) | 85 (30%) | 47 (39%) | 0.13 |
| No vehicles (%) | 37 (9%) | 21 (8%) | 16 (13%) | 0.17 |
| Group quarters (%) | 17 (4%) | 9 (3%) | 8 (7%) | 0.20 |
